# The epigenetic clock as a predictor of disease and mortality risk: a systematic review and meta-analysis

**DOI:** 10.1186/s13148-019-0656-7

**Published:** 2019-04-11

**Authors:** Peter D. Fransquet, Jo Wrigglesworth, Robyn L. Woods, Michael E. Ernst, Joanne Ryan

**Affiliations:** 1 Department of Epidemiology and Preventive Medicine, Monash University, ASPREE, Level 5, The Alfred Centre, 99 Commercial Road, Melbourne, Victoria 3004 Australia; 2 Disease Epigenetics, Murdoch Childrens Research Institute, The University of Melbourne, Parkville, Victoria 3052 Australia; 3 INSERM, U1061, Neuropsychiatrie, Recherche Clinique et Epidémiologique, Neuropsychiatry: Research Epidemiological and Clinic, Université Montpellier, 34000 Montpellier, France; 4 Department of Pharmacy Practice and Science, College of Pharmacy, The University of Iowa, Iowa City, IA USA; 5 Department of Family Medicine, Carver College of Medicine, The University of Iowa, Iowa City, IA USA

**Keywords:** Ageing, Age-related disease, Biological age, DNA methylation, Epigenetic clock, Longevity, Mortality, Systematic review

## Abstract

**Background:**

Ageing is one of the principal risk factors for many chronic diseases. However, there is considerable between-person variation in the rate of ageing and individual differences in their susceptibility to disease and death. Epigenetic mechanisms may play a role in human ageing, and DNA methylation age biomarkers may be good predictors of age-related diseases and mortality risk. The aims of this systematic review were to identify and synthesise the evidence for an association between peripherally measured DNA methylation age and longevity, age-related disease, and mortality risk.

**Methods:**

A systematic search was conducted in line with the Preferred Reporting Items for Systematic Reviews and Meta-Analyses (PRISMA) guidelines. Using relevant search terms, MEDLINE, Embase, Cochrane Central Register of Controlled Trials, and PsychINFO databases were searched to identify articles meeting the inclusion criteria. Studies were assessed for bias using Joanna Briggs Institute critical appraisal checklists. Data was extracted from studies measuring age acceleration as a predictor of age-related diseases, mortality or longevity, and the findings for similar outcomes compared. Using Review Manager 5.3 software, two meta-analyses (one per epigenetic clock) were conducted on studies measuring all-cause mortality.

**Results:**

Twenty-three relevant articles were identified, including a total of 41,607 participants. Four studies focused on ageing and longevity, 11 on age-related disease (cancer, cardiovascular disease, and dementia), and 11 on mortality. There was some, although inconsistent, evidence for an association between increased DNA methylation age and risk of disease. Meta-analyses indicated that each 5-year increase in DNA methylation age was associated an 8 to 15% increased risk of mortality.

**Conclusion:**

Due to the small number of studies and heterogeneity in study design and outcomes, the association between DNA methylation age and age-related disease and longevity is inconclusive. Increased epigenetic age was associated with mortality risk, but positive publication bias needs to be considered. Further research is needed to determine the extent to which DNA methylation age can be used as a clinical biomarker.

**Electronic supplementary material:**

The online version of this article (10.1186/s13148-019-0656-7) contains supplementary material, which is available to authorized users.

## Background

The population is ageing [1, 2], and age is one of the strongest risk factors for many human diseases, such as cardiovascular, metabolic and neurological diseases, and cancer [3]. This increased burden represents a major societal, economic, and public health challenge. Individuals, however do not all age to the same extent. There is considerable between-person variation in the rate of ageing, and individual differences in their susceptibility to disease and death. The identification of individuals at greatest risk of age-related diseases and death would provide important opportunities for targeting prevention and intervention.

There is thus great interest in molecular targets as clinical biomarkers which accurately predict the risk of age-related diseases and mortality. These biomarkers, which include cellular senescence, genomic instability, telomere attrition, and mitochondrial dysfunction, appear to capture pivotal aspects of biological age [4] and have been associated with a number of age-related diseases and mortality.

It is well established that as individuals age, there is a raft of molecular changes that occur within the cells and tissues. Changes in DNA methylation patterns have been shown to occur with ageing [5] and thus may be a fundamental mechanism that drives human ageing [6]. Epigenetic biomarkers of ageing, otherwise known as the epigenetic clock, have been developed using DNA methylation measurements. Referred to specifically as ‘DNA methylation age’ (DNAmAge), they provide an accurate estimate of age across a range of tissues, and at different stages of life [7, 8], and are some of the most promising biomarkers of ageing [9, 10]. DNAmAge has also permitted the identification of individuals who show substantial deviations from their actual chronological age, and this ‘accelerated biological aging’ has been associated with unhealthy behaviours [11], frailty [12], cancer [13], diabetes [14], cardiovascular diseases (CVD) [15], dementia [16], and mortality risk [17].

In the last few years, two meta-analyses of 13 studies (*n* = 13,089) and 4 studies (*n* = 4658), respectively, have been undertaken to investigate the extent to which DNAmAge in blood predicts mortality risk [17, 18]. Both reported a significant association between increased DNAmAge and mortality risk. However, neither was undertaken as part of a systematic review, raising the possibility that the findings were not representative of all research that has been undertaken in the field. To date, there has also been no systematic review that has investigated whether DNAmAge biomarkers are predictors of age-related diseases or longevity.

The aim of this systematic review is to identify and synthesise the evidence for an association between DNAmAge measured in peripheral tissues (blood, saliva, buccal cells), and longevity, age-related disease, and mortality risk.

## Methods

This systematic review protocol was registered as number CRD42018108568 on the international website for systematic reviews, PROSPERO (the International Prospective Register of Ongoing Systematic Reviews) [19]. The Preferred Reporting Items for Systematic Reviews and Meta-Analyses (PRISMA) guidelines (http://www.prisma-statement.org) [20, 21] were closely adhered to in the preparation of this systematic review.

### Inclusion criteria

#### Types of studies and participants

Cross-sectional studies, prospective cohorts, and case-control studies were eligible for inclusion in this review. Studies involving humans of any age, gender, race and ethnicity, and who were recruited from either the general community or a specific patient group, were eligible for inclusion. Animal studies, in vitro, and in vivo experiments were excluded.

#### Epigenetic clock (DNA methylation age)

Studies were eligible for inclusion in this systematic review if they extracted DNA from peripheral biological samples (blood, saliva, buccal swabs) and measured DNA methylation.

Studies met our eligibility criteria if they assessed DNAmAge with at least one of the two most widely used and well-validated epigenetic clocks; the Horvath clock [8] and Hannum’s clock [7]. The Horvath estimator is based on DNA methylation at 353 cytosine-phosphate-guanine base pairs (CpGs) [22]. The Hannum estimator is based on DNA methylation at 71 distinct CpGs.

To ascertain whether participants are biologically older or younger compared to their actual age, age acceleration (AA) is measured. This is done by determining the difference between an individual’s DNAmAge and their chronological age.

There are also some more recent variations to the AA measurements. Specifically, ‘intrinsic epigenetic age acceleration’ (IEAA) takes into account measures of blood cell counts and adjusts for this accordingly [23]. This provides a measure of AA independent of changes in blood cell composition, which can occur with age [24] or in response to immune system functions [23].

Another measure is ‘extrinsic epigenetic age acceleration’ (EEAA) [18], which incorporates the changes in cell composition by using a weighted average of age-associated cell counts. It thus provides a measure of AA that incorporates changes in age-related cell composition.

The eligible estimates of DNAmAge acceleration that were included in this review were thus:

1. Age acceleration calculated with Horvath’s clock (AAH)
2. Age acceleration calculated with Hannum’s clock (AAHa)
3. Intrinsic epigenetic age acceleration calculated with Horvath’s clock (IEAAH)
4. Intrinsic epigenetic age acceleration calculated with Hannum’s clock (IEAAHa)
5. Extrinsic epigenetic age acceleration calculated with Hannum’s clock (EEAA)

Studies using publicly available DNA methylation data were also included if they fit the other inclusion criteria.

#### Outcome measures and timing

This systematic review included studies focusing on age-related diseases (of any type), mortality, and longevity. Studies measuring associations with age-related disease, either tracked disease incidence in individuals initially free of the disease (and when DNAmAge was assessed), or compared DNAmAge between groups based on the presence or absence of disease (case-control study). Studies were excluded if they only measured the risk factors for age-related diseases (i.e. hypertension, hypercholesterolemia, obesity). We also included studies that investigated all-cause or cause-specific mortality and any studies which specifically looked at longevity.

#### Search strategy

A systematic search was conducted to identify relevant articles published through 2 September 2018, using the following databases: MEDLINE, Embase, Cochrane Central Register of Controlled Trials, and PsychINFO. Search terms included [epigenetic clock or epigenetic ag* or methylation ag* or (biological ag* and methyl*)] and [blood or serum or plasma or peripheral or leukocyte or PBMC* or mononuclear or buccal or saliva] and [longevity or mortality or death* or disease* or condition*]. A grey literature and Google Scholar search were also performed. Additional studies were identified by searching the reference list of the review articles identified from the database search, as well as those of the included studies. Studies that were published in either English or French were eligible for inclusion.

#### Synthesis of the data

After removal of duplicate articles, the title and abstracts were screened independently by two authors (JR and JW) to assess initial eligibility. The full text of any seemingly eligible article was then obtained, and suitability for inclusion in the systematic review was again assessed. Data was extracted independently by three authors (JR, JW, and PF) on a form developed specifically for this review and included information about the study design, location, and sample characteristics; the biological sample that was available, how DNA methylation was measured, and the calculator used to determine DNAmAge and AA; as well as the main findings from the study and any adjustment that was used in the analysis. Any discrepancies were resolved through discussion or consultation with a third author.

After having assessed the clinical and methodical heterogeneity, studies were grouped according to common outcome assessments. Where studies were considered clinically homogenous, and measured mortality outcomes, a meta-analysis was performed. For outcomes which were clinically too heterogenous, results are summarised quantitatively in tables and via a narrative synthesis, grouped according to types of outcomes.

Estimates of effect size were reported as correlations or beta values and standard errors from a linear regression for longevity outcomes, as odds ratios and 95% confidence intervals (95% CI) for dichotomous outcomes, or as hazard ratios (HR) and 95% CI for time-to-event (disease or mortality).

#### Methodological quality assessment

The papers that were included in the systematic review were all assessed for methodological quality using the Joanna Briggs Institute (JBI) Critical Appraisal Checklist for Cohort Study or Case-Study, as deemed appropriate [25]. For each study, the criteria listed on the checklist were rated as having a low, unclear, or high risk of bias. The risk of bias evaluation was used to help evaluate the quality of evidence from each study but not to exclude any studies from the review. This assessment was undertaken independently by two authors (JW and JR).

#### Meta-analysis

Review Manager (RevMan) 5.3 software [26] was used for a meta-analysis. Studies which were included needed to have reported HR and corresponding standard errors, or 95% confidence intervals. The natural log of HR and standard errors (some of which were calculated from confidence intervals), were calculated for each study independently, then pooled and weighted by generic inverse variance to provide an overall HR, 95% confidence interval, and *p* value. The *I*^2^ statistic (i.e. the percentage of variability between study outcomes), the chi-squared statistical test, and the corresponding *p* value were determined automatically by the programme and displayed in a forest plot.

## Results

### Search results

**Fig. 1:**
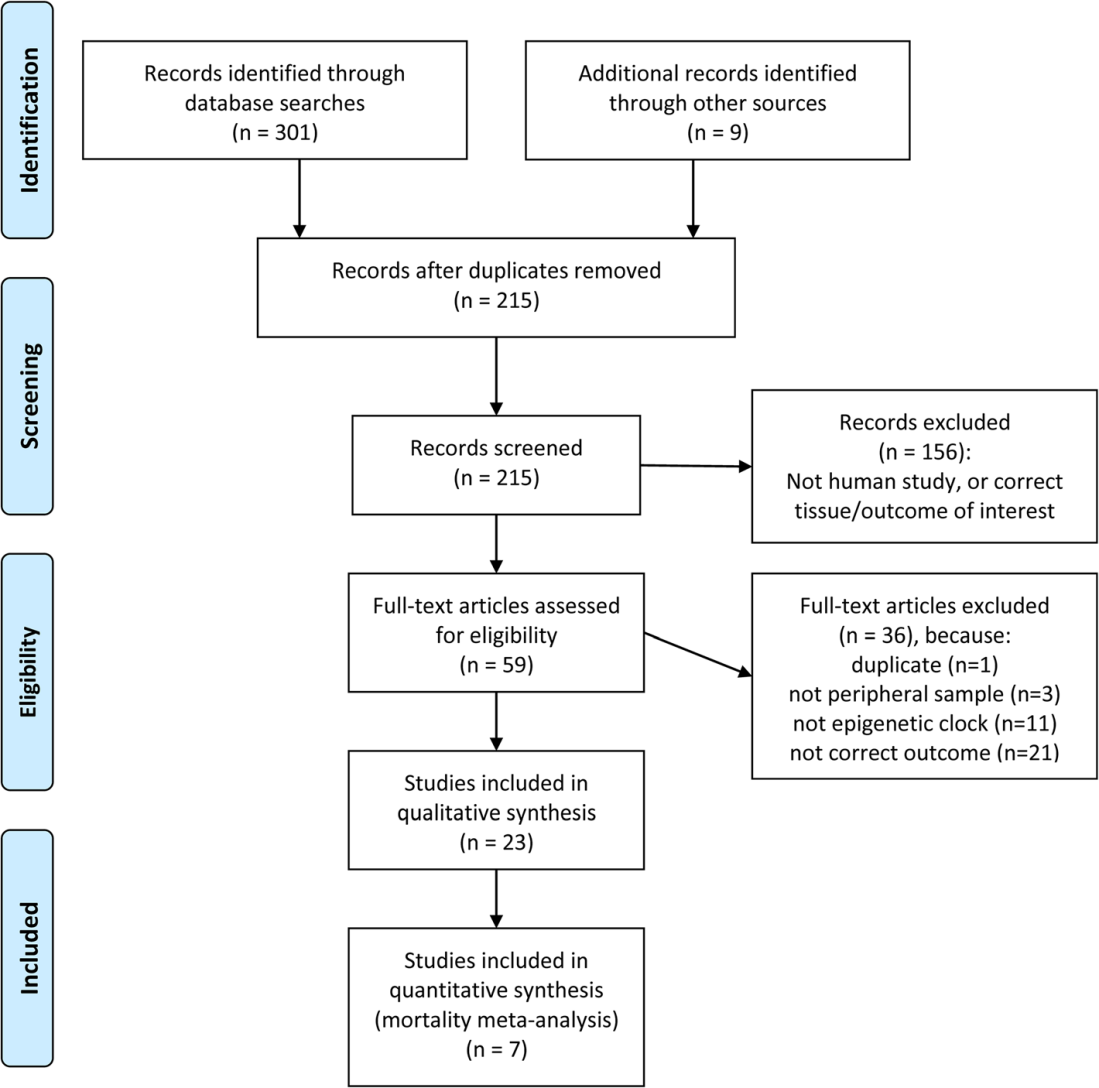
Prisma flow diagram

After duplicates were removed, 215 articles were identified from the search (Fig. 1) [27].

On inspection of titles and abstracts, 156 articles were excluded as they either did not measure DNAmAge in a peripheral sample in humans and/or did not investigate an appropriate outcome of interest. We selected 59 articles for full-text assessment.

Of these, three articles were excluded because they did not measure DNA methylation in a peripheral tissue [28–30] and 11 because they did not calculate DNAmAge using the epigenetic clock calculators that met our inclusion criteria [6, 31–40]. A further 21 studies were excluded because they did not measure an appropriate outcome. For example, 14 did not measure a disease outcome, mortality, or longevity [41–54], 1 study investigated prevalent, not incident, diabetes [55], 2 studies examined physical frailty [44, 56], 3 studies focused on ageing-related genetic conditions [57–59] and 1 on twin differences [60]. Finally, one study was excluded because it was subsequently found to be a duplicate [61].

### Characteristics of included studies

A total of 23 articles were included in this systematic review, involving 41,607 participants. For each study, we report pertinent characteristics of the study design, characteristics of the participants included, information regarding the calculation of DNAmAge, and the outcomes measured (Tables 1, 2, and 3).

**Table 1:** Studies investigating the association between the epigenetic clock and ageing, perceived age, or longevity

Paper	Study (country)	Characteristics: *n*, mean (SD) age, sex	Outcome	Sample; platform	Clock*	DNAmAge associations [correlation with chronological age]	Adj.
[65]	Study of semi-super centenarians (SSC), from three cities. Case-control (Italy)	75 SSC, 106 (NS), 79% ♀; 63 SSC offspring, 72 (NS), 40% ♀; 46 controls, 70 (NS), 80% ♀	Longevity	PBMCs; 450 K	AAH IEAAH EEAA	↓ 5.1 years SSC offspring vs. controls (*p* = 0.0004, significant AAH and IEAAH). EEAA NS. ↓ 8.6 years SSC vs. their age (β 6.58, *p* = 0.03) but NS vs. offspring [EAH assumed, *r* = 0.89]	Age, sex, cell %
[73]	Case-control YFS (25 years FU) V90+ (4 years FU) (Finland)	YFS: 49, 15 (NS), 59.2% ♀; 44, 18 (NS), 68.2% ♀; 55, 21 (NS), 56.4% ♀; 35, 24 (NS), 60.0% ♀. V90+: 122, 90 (NS), 73% ♀; 21 CT, aged 19–29, 66% ♀	Ageing	Blood (BL/FU); 450 K	EAH	YFS: ∆ EAH over 25 years relatively stable (*r* = 0.54, *p* < 0.001); V90+: ↑∆ EAH over 4 years in younger versus older individuals (7 vs. 4 years, *p* = 0.02). [*r* = 0.59–0.79, depending on sex and cohort]	Age, sex
[71]	Cohort Study SCS, OATS, BSGS (Australia)	275 Total, aged 34 to 103. SCS: 23, 97.9 (1.9), 47.8% ♀; OATS: 113, 70.4 (5.5), 61.9% ♀; BSGS: 139, 46.6 (5.6), 52.5% ♀	Ageing	Blood; 450 K	All	No correlation between DNAmAge separate studies SCS: ↓ AAHa but ↑ AAH compared to OATS and BSGS. No sig. between cohorts on IEAAH, IEAAHa and EEAA . [EAH *r* = 0.93, EAHa *r* = 0.96]	None
[66]	Case-control CRELES (Costa Rica)	Case-control sub-sample: 48 Nicoya, age 83 (14), 57% ♀; 47 non-Nicoya, age 85 (16), 55% ♀	Longevity	Whole blood; 450 K	EAH EAHa	No ∆ EAH/EAHa between groups (Nicoyans vs. non-Nicoyans) [EAH *r* = 0.86, EAHa *r* = 0.85]	Age

*AAH*, age acceleration (Horvath); *AAHa*, age acceleration (Hannum); *BL*, baseline; *BSGS*, Brisbane Systems Genetics Study; *CRELES*, Costa Rican Study on Longevity and Healthy Ageing; *CT*, controls; *DNAmAge*, DNA methylation age; *EEAA*, extrinsic epigenetic age acceleration (Hannum measurement); *EAH*, epigenetic age (Horvath); *EAHa*, epigenetic age (Hannum); *FU*, follow-up; *IEAAH*, intrinsic epigenetic age acceleration (Horvath); *IEAAHa*, intrinsic epigenetic age acceleration (Hannum); *NS*, not shown; *OATS*, Older Australian Twin Study; *SCS*, Sydney Centenarian Study; *SSC*, semi-super centenarians; *V90+*, Vitality 90+; *YFS*, Young Finns Study

*All includes all five measures of interest to this review, including AAH, AAHa, IEAAH, IEAAHa, and EEAA

**Table 2:** Studies investigating the association between the epigenetic clock and the incidence of age-related diseases

Paper	Study (country)	Characteristics: *n*, mean (SD) age, sex	Outcome	Sample; platform	Clock*	DNAmAge associations [correlation with chronological age]	Adj.
[69]	Cohort study WHI (USA)	2029, age 65.3 (7.1) 100% ♀ (post-menopause). 20 years FU	Lung cancer incidence; *n* = 43	Blood; 450 K	IEAAH	↑ risk of lung cancer (HR 1.50; *p* = 0.003), especially septuagenarians (HR:2.51, *p* = 0.0008) and current smokers (HR 6.17, *p* = 0.0004) [*NS*]	Age, ethnicity, smoking, and pack years
[70]	Cohort study NAS (USA)	442, 71.7 (6.7), 0% ♀, 370 seen pre 2003, 306 in cohort 2003 to 2013. 3–5 years FU	Cancer incidence, *n* = 132	Buffy coat (3 years FU); 450 K	IEAAHa	↑ cancer incidence (HR 1.06, *p* = 0.004 per year) in 2003–2013 cohort only [*NS*]	Age, BMI, education, smoking, alcohol
[63]	Case-control EPIC (10 countries)	902 Total, 52.3 (8.9), 100% ♀ 451 cases 451 CT	Breast cancer	Blood; 450 K	IEAAH	↑ breast cancer (4%, *p* = 0.016). In stratified analysis, only significant for postmenopausal ♀ [0.76]	BMI, hormonal factors, various others
[64]	Case-control EPIC (10 countries)	845 Total, 52 (7.4), 77.8% ♀ 235 BC 166 CRC	Breast/colorectal cancer	Blood; 450 K	AAH AAHa IEAAH IEAAHa	In males, ↑ AAH in CRC (1.6 years older, *p* = 0.04) NS all in females [*NS*]	Cell %
[13]	Seven case-control studies nested in MCCS Case-control (Australia)	6432 total, 27–76, 59% ♀ 3216 cancer cases** 3216 CT Median 8.3 years FU	Cancer	Blood; 450 K	All	↑ cancer risk (7 of 35 associations, *p* < 0.05, mainly kidney ↑ 35–63% and B cell lymphomas ↑15–27%). [EAH: *r* = 0.73, EAHa:0.78]	Age and sex, matched, various others
[23]	10 cohorts (∆ ethnicities) WHI, BHS, PEG (USA), cohorts from Bolivia, Asia, and Africa	Total 4296 (individual cohorts 41–1462), 2–92 (NS), between 0% and 100% ♀	Incident coronary heart disease (CHD), *n* = not stated	Blood (3× saliva) 450 K (2 × 27 K)	IEAAH EEAA	NS [EAH assumed: *r* = 0.65 to 0.93 depending on cohort, 3 of 10 > 0.80]	Age, sex, cell %, education
[62]	Cohort study IS: BASICMAR CT: REGICOR (Spain)	IS: 82, 63.9 (10.3), 45.1% ♀ CT: 41, 62.8 (14.2), 48.8% ♀	Ischemic stroke	Whole blood; 450 K	AAH AAHa	↑ stroke patients (AAHa: 2.5 years, *p* = 0.008; AAH NS), especially younger patients [All samples, EAH: *r* = 0.87, EAHa:0.80]	Sex
[67]	Two cohorts of ischemic stroke (IS) patients (Spain)	Discovery: 551, 65–81 (NS), 41.9% ♀; Replication: 85, 66–80 (NS), 29.4% ♀	IS outcome at 3 months (from 0 = no symptoms to 6 = death)	Whole blood; 450 K	AAHa	↑ worse 3-month stroke outcome (OR:1.04, *p* = 0.008 and OR 1.16, *p* < 0.001 in 2 cohorts). [*r* = 0.81]	Age, sex, smoking, treatment, various others
[74]	Cohort study PIVUS (Sweden)	832, 70 (NS), 50% ♀ 10years FU	Incident CVD, *n* = 153	Whole blood; 450 K	AAH AAHa	↑ incident CVD (3.3% per year, *p* = 0.02) with AAH. AAHa NS [*NS*]	Sex, smoking, BMI, various others
[16]	Case-control PEG (USA)	Caucasians: 289 PD, 37–91 (NS), 43% ♀; 219 CT, 35–92 (NS), 47% ♀. Hispanics: 46 PD, 37–83 (NS), 30% ♀ 38 CT, 35–92 (NS), 53% ♀	Parkinson’s disease (PD)	Blood; 450 k	AAH IEAAH EEAA	Associated with PD after logistic regression (AAH: *p* = 0.037; EEAA *p* = 0.031. Effect sizes not stated. [Caucasians: *r* = 0.82, Hispanics: *r* = 0.81]	Age, sex, cell %, smoking, ethnicity, coffee, pesticides
[68]	Cohort study Betula (Sweden)	16 maintain memory, 57.8 (3.6) 20 average, 58.0 (3.5) 16 decline, 57.9 (3.6), 50% ♀. 15 years FU	Dementia incidence, *n* = 7	Blood; 450 K	AAH	↑ dementia incidence (β 0.16, *p* = 0.03). [*r* = 0.69]	Age, sex

*AAH*, age acceleration (Horvath); *AAHa*, age acceleration (Hannum); *BASICMAR*. Abbreviation: *NS* (Ministerio de Sanidad y Consumo, Instituto de Salud Carlos III); *BC*, breast cancer; *Betula*, longitudinal cohort study of memory, health and ageing; *BHS*, Bogalusa Heart Study; *BL*, baseline; *CRC*, colorectal cancer; *CT*, controls; *CVD*, cardiovascular disease; *DNAmAge*, DNA methylation age; *EEAA*, extrinsic epigenetic age acceleration (Hannum measurement); *EAH*, epigenetic age (Horvath); *EAHa*, epigenetic age (Hannum); *EPIC*, European Prospective Investigation into Cancer and Nutrition; *FU*, follow-up; *IEAAH*, intrinsic epigenetic age acceleration (Horvath); *IEAAHa*, intrinsic epigenetic age acceleration (Hannum); *IS*, ischemic stroke; *MCCS*, Melbourne Collaborative Cohort Study; *NAS*, US Department of Veterans Affairs’ Normative Ageing Study; *NS*, not shown; *PEG*, Parkinson’s disease, Environment, and Genes case-control study; *PIVUS*, Prospective Study of the Vasculature in Uppsala Seniors; *REGICOR*, Registre Gironi del Cor; *WHI*, Women’s Health Initiative (WHI)

*Includes all five measures of interest to this review, including AAH, AAHa, IEAAH, IEAAHa, and EEAA

**CRC, gastric, kidney, lung, prostate, urothelial, B cell lymphoma

**Table 3:** Studies investigating the association between the epigenetic clock and mortality

Paper	Study (Country)	Characteristics: *n*, mean age (SD), sex	Outcome, deaths	Sample; platform	Clock*	DNAmAge associations [correlation with chronological age]	Adj.
[17]	Meta-analysis of 4 cohorts: LBC1921 and LBC1936 (Scotland), FHS and NAS (USA)	LBC1921: 446, age 79.1 (0.6), 60% ♀; LBC1936: 920, age 69.5 (0.8), 49% ♀; FHS: 2635, age 66.3 (8.9), 54% ♀; NAS: 657, age 72.9 (6.9), 0% ♀. 2–15 years FU	All-cause mortality, *n* = 862	Blood; 450 K	IEAAH^1^ IEAAHa^1^	16% and 9% ↑ mortality risk for a 5-year higher IEAAHa and IEAA respectively (*p* < 0.05). [EAH: *r* = 0.75, EAHa:0.83]	Chronological age, sex, smoking, education, childhood IQ, social class, hypertension, diabetes, cardiovascular disease, and APOE status.
[18]	Meta-analysis of 12 cohorts: WHI (× 3), NAS, ARIC, FHS, BLSA (USA); LBC1921 and 1936 (Scotland); KORA (Germany); InCHIANTI (Italy); Rotterdam (Netherlands)	Overall 12,284. Age range 52–79, % ♀ not stated. From 4 to 21 years FU	All-cause mortality, *n* = 2704	Blood; 450 K	AAH AAHa IEAAH EEAA	All measures of age acceleration are significantly associated with ↑ mortality risk (*p* ≤ 5.4e-05). EEAA outperformed all measures with the smallest *p* value. [EAH: *r* = 0.15–0.87, EAHa: *r* = 0.13–0.89, all depending on cohort, 10 of 26 > 0.80]	Age, BMI, various others
[72]	Twin study LSADT (Denmark)	LSADT: 86 same-sex twins, aged 73–82, 72% ♀	All-cause mortality *n* = 55	Blood; 450 K	AAH AAHa	35% ↑ mortality risk per 5-year ↑ in AA-Horvath (P = 0.02). 2-fold ↑ mortality risk for the twin with the oldest biological age. AAHa NS [EAH: *r* = 0.97]	Age, sex, cell composition, twin pairing.
[75]	Cohort study LBC1921 and LBC1936 (Scotland)	LBC1921: 414, age 79.1 (0.6), 58% ♀, 13 years FU LBC1936: 920, age 69.5 (0.8), 49%♀, 6 years FU	All-cause mortality, *n* = 280 and 135	Whole blood; 450 K	AAHa	25% ↑ mortality risk for each 1 SD ↑ in AAHa (*p* = 0.0001) (Pooled result) [*NS*]	Age (at baseline), sex
[76]	Cohort Study Louisiana Healthy Ageing Study Cohort (USA)	262, age 86 (10), 60.7% ♀, all Caucasian. Average 4.4 years FU	All-cause mortality, *n* = 206	Blood; 450 K	AAH	NS [*r* = 0.63]	Age, cell %
[77]	Cohort Study NAS (USA)	241, age 52.6 (10.7), 13% ♀. White non-Hispanic. 6.5 years FU, average 3.4 years	All-cause mortality, *n* = 17	Buffy coat; 450 K	AAHa	13% ↑ mortality risk over FU (*p* = 0.03). [*r* = 0.90]	Age (baseline), sex, cell %, PTSD, ancestry
[11]	Seven case-control studies nested in MCCS Case-control(Australia)	2818 healthy controls matched to cancer cases, age 27–76, 39% ♀. Median 10.7 years FU	All-cause mortality, *n* = 831; cancer, *n* = 240; CVD, *n* = 203; other-cause mortality, *n* = 249.	Blood; 450 K	All	5–8% ↑ risk of all-cause mortality per 5-year ↑ Horvath (both predictor types). 11–14% ↑ risk of cancer-related mortality (all predictors). NS with the risk of CVD or other-cause mortality (all predictors). [EAH: *r* = 0.73, EAHa:0.76]	Age, various others
[13]	Seven case-control studies nested in MCCS Case-control(Australia)	3216 cancer cases (different types) and controls, 27–76, 59% ♀. Median 8.3 years FU	All-cause mortality, *n* = 1726; cancer-related, *n* = 1271; other causes of death, *n* = 309.	Blood; 450 K	All	4–14% ↑ risk of all-cause, cancer-related and other causes of death per 5-year ↑ Hannum (all 3 predictors). Horvath NS predictive of mortality (all measured types). [EAH: *r* = 0.73, EAHa:0.78]	Age and sex, matched. Various others
[70]	Cohort study NAS (USA)	442 males, age 71.7 (6.7), 370 seen pre-2003, 306 in cohort 2003 to 2013. 3–5 year FU	Cancer mortality, *n* = 34	Buffy coat (3 years FU); 450 K	IEAAHa	17% ↑ cancer mortality per year (*p* = 0.001) in 2003–2013 cohort only. [*NS*]	Age at first blood draw, BMI, education, smoking, alcohol, and top 3 principal components
[78]	Cohort study ESTHER (Germany)	1863 total, aged 62.5 (6.6). 1260 survivors, 57.2% ♀, 602 cases (316 selected, 43.7% ♀ and 286 deceased in sub-sample, 39.5% ♀). Maximum 13 years FU	All-cause mortality, *n* = 602; cancer-causing mortality, *n* = 235; CVD-related mortality, *n* = 194.	Blood, baseline; 450 K	AAH AAHa	22–23% ↑ risk of all-cause and cancer-causing mortality per 5-year ↑ in AA-Horvath (*p* < 0.05). AA-Horvath NS with CVD mortality when the model was fully adjusted. NS AA-Hannum with any of the 3 measures of mortality [*r* = 0.77, 0.73) [EAH: *r* = 0.73, EAHa: *r* = 0.77]	Age, sex, BMI, various others
[67]	Two cohorts of ischemic stroke (IS) patients (Spain)	Discovery: 551, 65–81 (NS), 41.9% ♀; replication: 85, 66–80 (NS), 29.4% ♀	IS outcome at 3 months (from 0 = no symptoms to 6 = death)	Whole blood; 450 K	AAHa	↑ worse 3-month stroke outcome (OR: 1.04, *p* < 0.001 and OR: 1.06, *p* = 0.007 in 2 cohorts). [*r* = 0.81]	Biological age, sex, recanalization treatment, and basal NIHSS, various others

*AAH*, Age Acceleration (Horvath); AAHa, age acceleration (Hannum); *CVD*, cardiovascular disease; *DNAmAge*, DNA methylation age; *EEAA*, extrinsic epigenetic age acceleration (Hannum Measurement); *EAH*, epigenetic age (Horvath); *EAHa*, epigenetic age (Hannum); *EPIC*, European Prospective Investigation into Cancer and Nutrition; *ESTHER*, epidemiological investigations of the changes of preventing, recognising early and optimally treating chronic diseases in an elderly population; *FHS*, Framingham Heart Study; *FU*, follow-up; *IEAAH*, intrinsic epigenetic age acceleration (Horvath); *IEAAHa*, intrinsic epigenetic age acceleration (Hannum); *IS*, ischemic stroke; *LBC1921*, Lothian Birth Cohort 1921; *LBC1936*, Lothian Birth Cohort 1936; *LSADT*, Longitudinal Study of Ageing Danish Twins; *MCCS*, Melbourne Collaborative Cohort Study; *NAS*, US Department of Veterans Affairs’ Normative Ageing Study; *NIHSS*, NIH Stroke Scale; *NS*, not shown; *WHI*, Women’s Health Initiative (WHI)

*Includes all five measures of interest to this review, including AAH, AAHa, IEAAH, IEAAHa, and EEAA

^1^Although not specifically stated, these measures of AA adjusted for cell type proportions in blood; hence both measures are intrinsic epigenetic age acceleration

### Study design and participants

There were 8 case-control studies with a total of 4671 cases and 7320 controls, including participants with Parkinson’s disease [16], ischemic stroke [62], and cancer [11, 13, 63, 64], as well as 2 studies of participants selected on the basis of their very old age [65, 66].

The remaining studies were prospective cohorts (*n* = 29,616 participants), which included three meta-analyses [17, 18, 23]. The follow-up time in these studies varied between 3 months [67] and 21 years [18]. Overall, the studies ranged in size between 52 [68] and 12,284 participants [18]. Participants were aged between 2 [23] and 106 years [65]. Most studies included both male and female participants; however, one used data from the Women’s Health Initiative (WHI) cohort of postmenopausal women [69], and one used data on only men from the US Department of Veterans Affairs’ Normative Ageing Study (NAS) [70].

### Risk of bias assessment

Eighty percent of the cohort studies (Additional file 1: Table S1) had a low risk of bias for all criteria, but only one of the eight case-control studies showed a low risk of bias (Additional file 1: Table S2) [25]. In the latter case, many of the studies provided inadequate information about whether the same criteria were used to identify cases and controls, and only half of the studies reported that cases and controls were matched. Across all study types, confounding factors were not clearly considered in the analysis of three [66, 71, 72], two studies did not recruit all individuals from the same source population [62, 71], and another two studies did not provide sufficiently clear information on source population [16, 73].

### Summary of outcomes

#### Longevity and ageing

Only four studies (*n* = 880) investigated differential DNAmAge and longevity or ageing (Table 1). Two studies investigated longevity specifically [65, 66] and found that DNAmAge was correlated with chronological age (*r* = 0.89, and *r* = 0.85–0.86 respectively). Their other findings, however, varied. Horvath et al. found that semi-super centenarians have a lower DNAmAge compared to chronological age (∆ − 8.6 years), and their offspring have a lower AAH and IEAAH compared to controls (but not compared to their chronological age) [65]. McEwan et al. found no age acceleration differences between long-lived Nicoyans and age-matched controls [66].

Another study investigated associations between DNAmAge and chronological age in three separate cohorts (including centenarians) [71]. When all three studies were combined, DNAmAge was highly correlated with chronological age (*r* = 0.93), but the correlation was much lower in each cohort separately (*r* = 0.52–0.73). The direction of AA was not concordant between AAH and AAHa measures across the three cohorts. The fourth study focused on long-term change in DNAmAge with ageing [73]. DNAmAge was moderately correlated with chronological age over the span of 25 years (*r* = 0.54). Younger participants aged faster (ageing seven DNA methylation years over four chronological years) than older participants.

#### Age-related disease

A total of 11 studies examined the association between DNAmAge and age-related diseases (Table 2). There were 5 studies of 10,650 participants that focused on cancer [13, 63, 64, 69, 70]. However, two separate studies used the same cohort, and some of the participants could have been the same [63, 64]. All studies found that increased DNAmAge (at least one of their measures) was associated with an increased risk of cancer incidence; however, the type of cancer and exact associations varied. For example, of the two studies which examined breast cancer [63, 64], only one reported a significant association [63], and the two studies of lung cancer also had discordant results [13, 69]. On the other hand, the two studies of colorectal cancer, reported very similar findings, with AAH positively associated with risk [13, 64].

There were four studies focusing on cardiovascular-related diseases including stroke and coronary heart disease [23, 62, 67, 74]. Two studies, authored by the same group, looked at different outcomes associated with ischemic stroke [62, 67]. The first found an association between increased DNAmAge and ischemic stroke (AAHa + 2.5 years, *p* = 0.008) [62], and the second showed that increased DNAmAge was associated with poorer outcomes 3 months post-stroke [67]. It is not clear if the same participants were included in both studies. A large study of 4296 individuals from 10 separate cohorts did not find any evidence of an association between DNAmAge and incident coronary heart disease [23]. However, a smaller (*n* = 832) more recent study found that for every 1-year increase in DNAmAge (AAH), there was a 3.3% greater incidence of cardiovascular disease (*p* = 0.02) [74].

The remaining two studies looked at dementia [16, 68] and reported findings in a similar direction. Younger DNAmAge was associated with better memory, and increased DNAmAge predicted incident dementia [68]. Both AAH and EEAA were also positively associated with Parkinson’s disease.

#### Mortality

Eleven studies (27,840 participants, 10,233 deaths) investigated the association between age acceleration and mortality (Table 3) [11, 13, 17, 18, 67, 70, 72, 75–78]. Results from 7 of the 11 studies, involving 17 individual population samples and 17,988 participants (5277 deaths), were combined to perform two independent meta-analyses (6 studies per epigenetic clock method) that investigated all-cause mortality [11, 17, 18, 72, 76–78].

Three of these studies [17, 72, 78] measured associations using unadjusted AA, whilst two studies measured associations with all (AA, IEAA, and EEAA), or otherwise all but one (IEAAHa) of the five variations of age acceleration, previously described. The two remaining studies were included in one of the two meta-analyses, focusing on the more common measures of DNAmAge, namely, AAH and AAHa [76, 77]. The HR and SE reported from a multivariate-adjusted Cox regression model for time to death (all-cause) were used for each study.

**Fig. 2:**
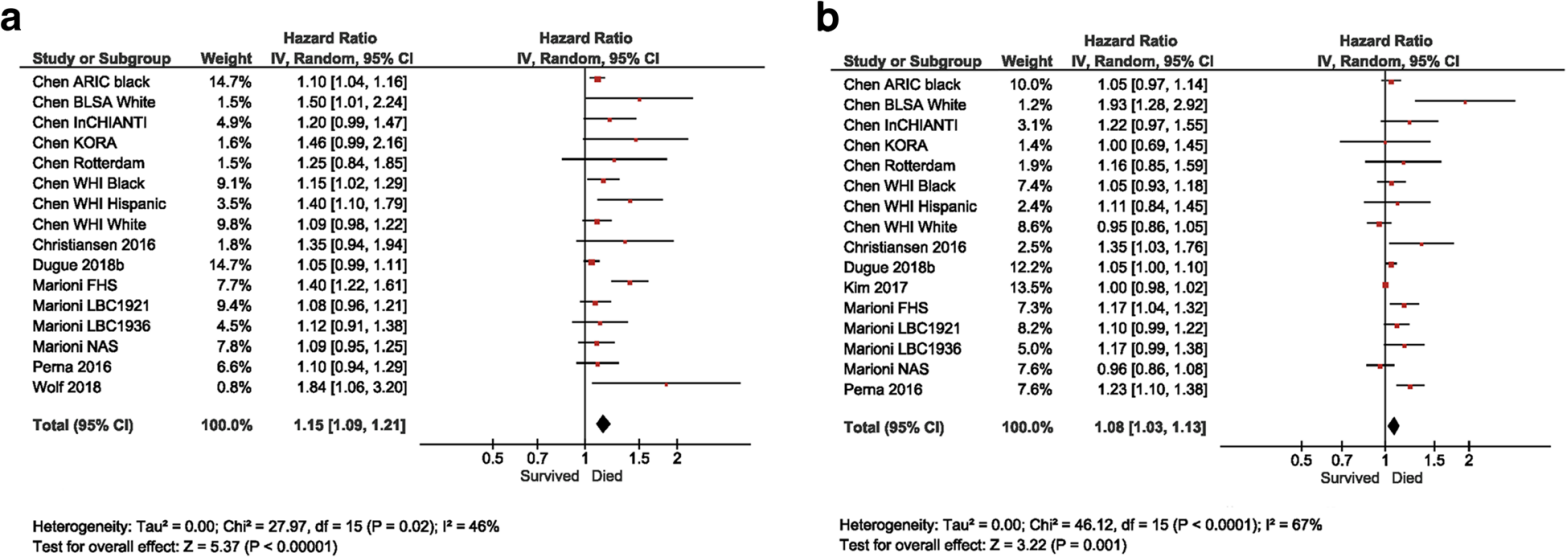
Forest plots for Horvath and Hannum meta-analyses. Meta-analyses used HR and standard errors collected from seven of the nine studies measuring associations between age acceleration for **a** AAHa and **b** AAH, and all-cause mortality. HR and 95% CI’s were calculated independently via a univariate Cox regression model and combined to provide a total value of risk. ARIC, Atherosclerosis Risk in Communities Studies; BLSA, Baltimore Longitudinal Study of Ageing; InCHIANTI, Invecchiare in Chianti, ageing in the Chianti area; KORA, Cooperative Health Research in the Augsburg Region; Rotterdam: The Rotterdam Study; WHI, Women’s Health Initiative; FHS, Framingham Heart Study; LBC1921, Lothian Birth Cohort 1921; LBC1936 Lothian Birth Cohort 1936; LSADT, NAS, US Department of Veterans Affairs’ Normative Ageing Study

Weighted average varied for each study and depended on the SE defined by the sample size, thus those with a larger sample size contributed the most to the resulting HR and 95% CI for each meta-analysis. Heterogeneity between and within studies was moderate to high (46% and 67% for AAHa and AAH respectively), and a random-effects model was thus used [79]. As presented in Fig. 2a, b, a higher biological age (per 5-year increase in age) was associated with an 8% and 15% increased risk of all-cause mortality for AAH and AAHa respectively.

**Fig. 3:**
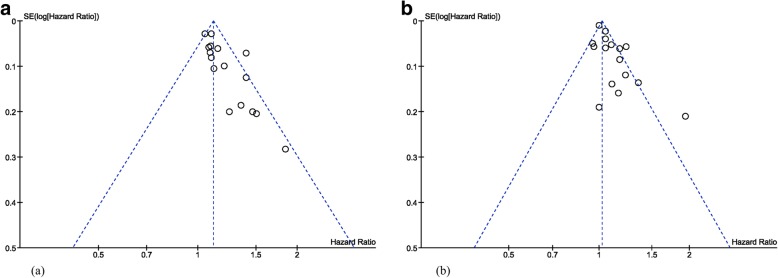
Funnel plots for Horvath and Hannum meta-analyses

The funnel plots for both measures were asymmetrical, indicating positive publication bias (Fig. 3). Results were similar for the two remaining studies not included in the meta-analysis [13, 75].

Four of the eleven studies [11, 13, 70, 78] (8339 participants, 1780 died), examined cancer-related deaths, with each reporting a significantly increased risk of cancer-related mortality in those with a higher DNAmAge. Effect sizes varied (ranging 4–23%), and there was no obvious pattern in relation to the duration or follow-up, nor study design (case-control vs. cohort only). This contrasts with findings from the three remaining studies (3896 participants, 477 deaths) investigating associations between AA and CVD, where a higher DNAmAge was only found to be significantly associated with an increased risk of mortality in those who had already experienced a CVD related event [67].

## Discussion

### Main findings

An increasing number of studies have investigated the association between DNAmAge, longevity, age-related disease, and mortality, with a total of 23 studies included in this systematic review and all published from 2015 onwards. Our primary finding is that there is sufficient evidence to support an association between accelerated DNAmAge, in particular for the Hannum epigenetic clock (AAHa), and an increased risk of all-cause mortality.

The majority of studies (10 out of 11) independently found that a higher biological age relative to chronological age is a predictor of time-to-death, cancer-related, CVD-related, or all cause. Of these studies, two stratified by sex to determine possible differential effects [17, 18], and two adjusted for sex using an interaction effect with biological age [11, 78]. Some other studies included only males [70], or females [18]. There was, however, no clear difference in the association between the epigenetic clock and the risk of death across the sexes. Likewise, findings from the two studies which considered ethnicity (as defined by country of birth or race) [11, 18], do not provide any evidence for differences between groups.

Collectively, these results are supported by our two meta-analyses for all-cause mortality. Interestingly, risk was greater when predicted by AAHa compared to AAH (15% vs. 8%, respectively), a finding supported by the two meta-analyses on this topic [17, 18]. It thus appears that these epigenetic calculators are measuring slightly different components of the ageing process. Indeed, it has been suggested that Horvath’s calculator is more suited for innate process that accompany development such as puberty and menopause, whilst Hannum’s may better reflect later-life diseases states and mortality [34]. Differences in the findings depending on the DNAmAge predictor used may also relate to how these algorithms were initially constructed. Specifically, Horvath’s epigenetic clock algorithm was developed as a robust multi-tissue age predictor based on DNA methylation at 353 CpGs, compared with Hannum’s epigenetic clock which is a blood-based estimator, defined by DNA methylation at 71 CpG sites [80].

There were 11 studies that investigated the association between DNAmAge and age-related disease. These showed that there is some evidence, although with often varying findings, that DNAmAge might be positively associated with the incidence of age-related diseases. It was difficult to make any disease-specific comparisons, as even within disease groups, the outcomes were highly heterogeneous. For example, the two studies concerning ischemic stroke investigated different outcomes, one being ischemic stroke incidence [62], and the other being the severity of ischemic stroke outcomes at a follow-up time point [67]. However, despite different study samples and investigating various outcomes, all but one of the studies found that increased DNAmAge predicted future risk of disease. These findings are also in concordance with those for mortality, and further support the potential of DNAmAge as a global biomarker of biological ageing and health.

Finally, the association between DNAmAge and longevity remains unclear, given that we identified only four eligible studies which were all relatively small (the largest *n* = 257). Comparisons of findings were not possible, as the scope of these studies were relatively broad, having very different study designs with unique sample characteristics. For example, one study focused on a sample from a Costa Nicoyan region of Costa Rica which is known as a hot spot of high longevity [81] and compared these individuals with non-Nicoyans. Nicoyans, however, may be ethnically different with very specific environmental exposures and lifestyle behaviours. In contrast, the other studies of longevity [65] or ageing [71, 73] compared small groups of individuals at various life stages, but who were selected from similar community populations. Future work in this field should focus on the study of centenarians or long-lived disease-free individuals as they may hold the answer to extended healthy lifespans. In understanding the underlying epigenetic mechanisms of ageing, such as altered DNA methylation patterns, and how it affects ageing-related genome maintenance, there is potential to directly promote healthy longevity, in turn possibly preventing age-related diseases [82].

### Quality and strength of the evidence

The JBI Critical Appraisal has shown that most studies in this review were not at risk of bias (57%). However, many studies did not report on, or were unclear about, the consistency of population, the matching of cases to controls, the selection criteria to identify cases and controls, or adjusting for possible confounding factors (43%) (Additional file 1: Tables S1 and S2). The omission of descriptions is possibly due to many studies using several already established cohorts.

Within the 23 studies, there were a total of 41,607 participants. Although some participants clearly do overlap between studies, it is not clear to what extent. Eight individual cohorts/studies (WHI, NAS, EPIC, MCCS, PEG, BHS, LBC1921, LBC1936) were used in more than one analysis, creating a possible bias in findings, and it is thus unclear whether these studies are using the same or similar data. For example, the US Department of Veterans Affairs’ Normative Ageing Study (NAS) was used in four separate studies [17, 18, 70, 77], and the Women’s Health Initiative (WHI) was used in three [18, 23, 69]. Further, two studies that looked at cancer as an outcome both used the same cohort (EPIC) and had similar sample sizes (*n* = 902 vs, *n* = 845), but it was not clear whether the data was overlapping between both studies [63, 64].

Whilst nearly every study, apart from one [23], showed some evidence of an association between at least one of the DNAmAge measures examined and an outcome, there were few studies which were directly replicated across more than one study. The variability in these associations may be related to the different DNAmAge measures which have been used, as well as the specific outcomes. For example, the five cancer studies looked at eight specific types of cancer [70], but different cancers are known to have very specific DNA methylation patterns [83]. Whether this could also directly influence DNAmAge, and potentially the accuracy of this biological age predictor, is unclear.

Despite evidence pointing towards an association with all-cause mortality, the centrality of studies, observed in both funnel plots (Fig. 3) is an indication of positive publication bias, and thus caution should be taken when interpreting these findings.

### Accuracy of age estimation

It has been suggested that to be an accurate biological age estimator, DNAmAge should be highly correlated with chronological age (*r* ≥ 0.80) (20 to 100 years) [80]. However, of the studies included in this systematic review, only 8 of the 23 studies reported a correlation between DNAmAge and chronological age at or above this level. Ten studies had either *r* < 0.80 on at least one measure of DNAmAge (either Horvath or Hannum) or had a lower correlation for all measures (*r* = 0.13–0.79). If DNAmAge is not highly correlated with chronological age, then the measures of age acceleration may also be less accurate. One of the reasons for this may be that most of the included studies focused on a narrow age range of older individuals, whilst the epigenetic clock algorithms were developed for individuals across a wide spectrum of ages (from 0 to 100 years). The lower correlations may also suggest that the measured DNAmAge of participants are being confounded by environmental factors beyond what studies have adjusted for. This is a particularly important point, given that DNA methylation levels are dynamic and may be influenced by environmental factors such as stress [84] and smoking [85].

### Strengths and limitations of the review

This systematic review was conducted in line with the Preferred Reporting Items for Systematic Reviews and Meta-Analyses (PRISMA) guidelines. A systematic search was established with clear inclusion and exclusion criteria, and for all studies included, the quality of evidence was evaluated. A meta-analysis was performed by pooling the data of multiple studies, giving greater certainty to the results. However, for both Horvath and Hannum methods, studies showed a moderate to high amount of heterogeneity, suggesting that studies were not undertaken in the same way or that different experimental protocols were applied. Heterogeneity may also be the result of including studies with varying cohorts, for example, the pooling of data from same-sex twins, combined with a male-only study, and a population study. As previously stated, funnel plots suggest that there was publication bias.

Limitations to our systematic review are that only studies assessing either the Horvath and/or Hannum epigenetic clocks were included, which are the most commonly used measures. However, there are a number of newer DNA methylation age estimators that have also been developed. For example, a recent ‘phenotypic age estimator’ was developed [34] which shows very good predictive accuracy for time to death in association with a number of markers of immunosenescence and smoking status.

It remains unclear whether these methylation changes at specific CpGs are driving ageing or are consequences of the ageing process (cellular ageing, underlying disease processes). Whilst larger sets of CpGs can produce more precise estimations of age [80], many measures in this review only showed modest or weak associations with chronological age. A meta-analysis could not be undertaken in regard to longevity or age-related disease as studies were too few and measures and outcomes too heterogeneous.

Whilst measures of biological age and their associations with mortality are more certain, the clinical practicality of measuring DNAmAge proves to be problematic, for example, when compared to physical tests that are also able to predict mortality, such as walking speed, grip strength, and BMI measurements, which are cheaper and far easier to obtain [86]. Should the cost of measuring DNAmAge come down significantly, it would be a viable measure of risk for all-cause mortality. These studies may provide practical suggestions for obtaining healthy longevity through the active modification of DNA methylation patterns by changing lifestyle habits. Both these, and focusing on modifying age-related, disease specific DNA methylation profiles may also aid in decreasing incidence of age-related disease or early mortality.

### Recommendations for future studies

In designing future studies in this field, some of the following points should be considered. Cohort studies are preferred over case-controls, with the latter being more susceptible to bias as we identified in this review. Cases and controls must be sampled from the same source population and sufficiently well matched. Thorough phenotyping of the study population more generally is also essential. This helps rule out competing exposures or diseases which may also confound the associations. Somewhat surprisingly, there was a lack of evidence for sex- or ethnic-specific effects observed in this systematic review, but future studies should also consider analysing and reporting this data individually. Longitudinal studies, that follow individuals over time and track disease progression, together with biological samples taken at several time points, would have the greatest value and could shed light on whether DNA methylation changes are driving ageing and age-related disease, or if they are the consequence of these processes.

In terms of reporting results, it is essential that studies provide comprehensive details relating to the participant’s characteristics, and present all data analysis that has been undertaken. Replication and validation of findings across multiple independent samples or cohorts are crucial. This will help reduce the reporting of false-positive findings.

Finally, the epigenetic clocks included in this review (Horvath and Hannum) were developed to measure biological age across a wide range of chronological ages. It could be that there is greater utility in developing an epigenetic clock specifically for later life that could encapsulate the lifetime exposure to a range of environmental factors and the increased prevalence of comorbidities. This may also be the period of the lifespan where predicting the risk of disease and mortality could be particularly pertinent in terms of interventions/treatments or prevention.

## Conclusion

Some measures of biological age presented in this systematic review may reflect longevity in long-lived individuals and risk of age-related disease. However, due to the relatively small number of studies and variability in findings, the evidence is as yet insufficient to confirm the utility of DNAmAge as a clinical biomarker in this regard.

DNAmAge is one of the most highly studied markers of ageing [87], and, with the limitations discussed here, appears to be a good predictor of mortality. An accurate measure of DNAmAge, that in theory could be measured at any age, has great potential to be an early biomarker of disease risk. Identifying individuals with accelerated biological ageing could permit targeted interventions to help delay their risk of age-related disease and increase their overall health. With the ageing population, there is increasing emphasis on promoting the health and well-being of older individuals. Given its importance, multiple studies into specific outcomes, with a wider assortment of study cohorts, should be explored further.

Given that DNA methylation is an epigenetic mechanism involved in gene regulation, beyond the ability to estimate future risk of disease and mortality, further studies could provide novel insights into the long-standing question about why and how people age. They may also offer answers as to how we may prevent the negative effects of ageing such as age-related diseases. Thus, it is of particular importance in future studies not only to measure DNAmAge, but also to investigate which interventions (e.g. lifestyle changes) attenuate the advancement or initiate the reversal of biological age directly.

## Additional files

Additional file 1: **Table S1.** Critical appraisal of cohort studies included in the review, using relevant criteria from the JBI Critical Appraisal Checklist [1]. **Table S2.** Critical appraisal of case-control studies included in the review, using relevant criteria from the JBI Critical Appraisal Checklist [1]. (DOCX 93 kb)

Additional file 2: Supplimentary MetaData from meta-analysis. (XLSX 12 kb)

## Abbreviations

AA: Age acceleration
AAH: Age acceleration calculated with Horvath’s clock
AAHa: Age acceleration calculated with Hannum’s clock
BHS: Bogalusa Heart study
CpG: Cytosine-phosphate-guanine
CVD: Cardiovascular diseases
DNAmAge: DNA methylation age
EEAA: Extrinsic epigenetic age acceleration calculated with Hannum’s clock
EPIC: European Prospective Investigation into Cancer and Nutrition
IEAAH: Intrinsic epigenetic age acceleration calculated with Horvath’s clock
IEAAHa: Intrinsic epigenetic age acceleration calculated with Hannum’s clock
JBI: Joanna Briggs Institute
LBC1921: Lothian Birth Cohort 1921
LBC1936: Lothian Birth Cohort 1936
MCCS: Melbourne Collaborative Cohort Study
NAS: US Department of Veterans Affairs’ Normative Ageing Study
PEG: Parkinson’s disease, Environment, and Genes case-control study
PRISMA: Preferred Reporting Items for Systematic Reviews and Meta-Analyses
WHI: Women’s Health Initiative
