# Supplementary material for: The epigenetic clock as a predictor of disease and mortality risk: a systematic review and meta-analysis
Source: Clin Epigenetics. 2019 Apr 11;11:62. doi: 10.1186/s13148-019-0656-7 (PMC6458841; doi:10.1186/s13148-019-0656-7)
Supplement: Supplementary file 1 — Table S1. Critical appraisal of cohort studies included in the review, using relevant criteria from the JBI Critical Appraisal Checklist [1]. Table S2. Critical appraisal of case-control studies included in the review, using relevant criteria from the JBI Critical Appraisal Checklist [1]. (DOCX 93 kb) [file 13148_2019_656_MOESM1_ESM.docx]

**Supplementary Table 1**. Critical Appraisal of Cohort Studies Included in the Review, using relevant criteria from the JBI Critical Appraisal Checklist [1].

| Reference | Citation | Groups from the same population | Exposure measured similarly for all | Exposure measured in a valid way | Strategies to deal with confounding | Valid measure of outcome | Appropriate statistics |
| --- | --- | --- | --- | --- | --- | --- | --- |
| Armstrong, 2017 | [2] | No | Yes | Yes | Unclear | Yes | Yes |
| Chen, 2016 | [3] | Yes | Yes | Yes | Yes | Yes | Yes |
| Christiansen, 2016 | [4] | Yes | Yes | Yes | No | Yes | Yes |
| Degerman, 2017 | [5] | Yes | Yes | Yes | Yes | Yes | Yes |
| Horvath, 2016 | [6] | Yes | Yes | Yes | Yes | Yes | Yes |
| Kananen, 2016 | [7] | Unclear | Yes | Yes | Yes | Yes | Yes |
| Kim, 2017 | [8] | Yes | Yes | Yes | Yes | Yes | Yes |
| Levine, 2015 | [9] | Yes | Yes | Yes | Yes | Yes | Yes |
| Lind, 2018 | [10] | Yes | Yes | Yes | Yes | Yes | Yes |
| Marioni, 2015 | [11] | Yes | Yes | Yes | Yes | Yes | Yes |
| Marioni, 2016 | [12] | Yes | Yes | Yes | Yes | Yes | Yes |
| Perna, 2016 | [13] | Yes | Yes | Yes | Yes | Yes | Yes |
| Soriano-Tarraga, 2017 | [14] | Yes | Yes | Yes | Yes | Yes | Yes |
| Wolf, 2018 | [15] | Yes | Yes | Yes | Yes | Yes | Yes |
| Zheng, 2016 | [16] | Yes | Yes | Yes | Yes | Yes | Yes |

Note: references are listed in alphabetical order

**Supplementary Table 2**. Critical Appraisal of Case-Control Studies Included in the Review, using relevant criteria from the JBI Critical Appraisal Checklist [1].

| Reference | Citation | Cases & controls from same population | Case & controls matched | Same criteria used to identify cases & controls | Valid exposure measure | Consider confounding | Valid outcome measure | Exposure time period sufficient | Appropriate statistics |
| --- | --- | --- | --- | --- | --- | --- | --- | --- | --- |
| Ambatipudi, 2017 | [17] | Yes | Yes | Yes | Yes | Yes | Yes | Yes | Yes |
| Dugue, 2018a | [18] | Yes | Yes | Unclear | Yes | Yes | Yes | Yes | Yes |
| Dugue, 2018b | [19] | Yes | Yes | Unclear | Yes | Yes | Yes | Yes | Yes |
| Durso, 2017 | [20] | Yes | Unclear | Yes | Yes | Yes | Yes | Yes | Yes |
| Horvath, 2015a | [21] | Unclear | No | Unclear | Yes | Yes | Yes | Yes | Yes |
| Horvath, 2015b | [22] | Yes | No | Yes | Yes | Yes | Yes | Yes | Yes |
| McEwen, 2017 | [23] | Yes | Yes | Yes | Yes | Unclear | Yes | Yes | Yes |
| Soriano-Tarraga, 2016 | [24] | No | Unclear | Unclear | Yes | Yes | Yes | Yes | Yes |

Note: references are listed in alphabetical order

**References**

1. The Joanna Briggs Institute. Joanna Briggs Institute Reviewers' Manual: 2014 Edition. Adelaide: The Joanna Briggs Institute; 2014.

2. Armstrong NJ, Mather KA, Thalamuthu A, Wright MJ, Trollor JN, Ames D, et al. Aging, exceptional longevity and comparisons of the Hannum and Horvath epigenetic clocks. Epigenomics. 2017;9(5):689-700.

3. Chen BH, Marioni RE, Colicino E, Peters MJ, Ward-Caviness CK, Tsai PC, et al. DNA methylation-based measures of biological age: meta-analysis predicting time to death. Aging (Albany NY). 2016;8(9):1844-65.

4. Christiansen L, Lenart A, Tan Q, Vaupel JW, Aviv A, McGue M, et al. DNA methylation age is associated with mortality in a longitudinal Danish twin study. Aging Cell. 2016;15(1):149-54.

5. Degerman S, Josefsson M, Nordin Adolfsson A, Wennstedt S, Landfors M, Haider Z, et al. Maintained memory in aging is associated with young epigenetic age. Neurobiol Aging. 2017;55:167-71.

6. Horvath S, Gurven M, Levine ME, Trumble BC, Kaplan H, Allayee H, et al. An epigenetic clock analysis of race/ethnicity, sex, and coronary heart disease. Genome Biol. 2016;17(1):171.

7. Kananen L, Marttila S, Nevalainen T, Kummola L, Junttila I, Mononen N, et al. The trajectory of the blood DNA methylome ageing rate is largely set before adulthood: evidence from two longitudinal studies. Age (Dordr). 2016;38(3):65.

8. Kim S, Myers L, Wyckoff J, Cherry KE, Jazwinski SM. The frailty index outperforms DNA methylation age and its derivatives as an indicator of biological age. Geroscience. 2017;39(1):83-92.

9. Levine ME, Hosgood HD, Chen B, Absher D, Assimes T, Horvath S. DNA methylation age of blood predicts future onset of lung cancer in the women's health initiative. Aging (Albany NY). 2015;7(9):690-700.

10. Lind L, Ingelsson E, Sundstrom J, Siegbahn A, Lampa E. Methylation-based estimated biological age and cardiovascular disease. European Journal of Clinical Investigation. 2018;48 (2) (no pagination)(e12872).

11. Marioni RE, Shah S, McRae AF, Chen BH, Colicino E, Harris SE, et al. DNA methylation age of blood predicts all-cause mortality in later life. Genome Biol. 2015;16:25.

12. Marioni RE, Harris SE, Shah S, McRae AF, von Zglinicki T, Martin-Ruiz C, et al. The epigenetic clock and telomere length are independently associated with chronological age and mortality. Int J Epidemiol. 2016;45(2):424-32.

13. Perna L, Zhang Y, Mons U, Holleczek B, Saum KU, Brenner H. Epigenetic age acceleration predicts cancer, cardiovascular, and all-cause mortality in a German case cohort. Clin Epigenetics. 2016;8:64.

14. Soriano-Tarraga C, Mola-Caminal M, Giralt-Steinhauer E, Ois A, Rodriguez-Campello A, Cuadrado-Godia E, et al. Biological age is better than chronological as predictor of 3-month outcome in ischemic stroke. Neurology. 2017;89(8):830-6.

15. Wolf EJ, Logue MW, Stoop TB, Schichman SA, Stone A, Sadeh N, et al. Accelerated DNA Methylation Age: Associations with Posttraumatic Stress Disorder and Mortality. Psychosomatic Medicine. 2018;80(1):42-8.

16. Zheng Y, Joyce BT, Colicino E, Liu L, Zhang W, Dai Q, et al. Blood Epigenetic Age may Predict Cancer Incidence and Mortality. EBioMedicine. 2016;5:68-73.

17. Ambatipudi S, Horvath S, Perrier F, Cuenin C, Hernandez-Vargas H, Le Calvez-Kelm F, et al. DNA methylome analysis identifies accelerated epigenetic ageing associated with postmenopausal breast cancer susceptibility. Eur J Cancer. 2017;75:299-307.

18. Dugue PA, Bassett JK, Joo JE, Jung CH, Ming Wong E, Moreno-Betancur M, et al. DNA methylation-based biological aging and cancer risk and survival: Pooled analysis of seven prospective studies. Int J Cancer. 2018;142(8):1611-9.

19. Dugue PAB, Bassett JK, Joo JE, Baglietto L, Jung CH, Wong EM, et al. Association of DNA Methylation-Based Biological Age With Health Risk Factors and Overall and Cause-Specific Mortality. Am J Epidemiol. 2018;187(3):529-38.

20. Durso DF, Bacalini MG, Sala C, Pirazzini C, Marasco E, Bonafe M, et al. Acceleration of leukocytes' epigenetic age as an early tumor and sex-specific marker of breast and colorectal cancer. Oncotarget. 2017;8(14):23237-45.

21. Horvath S, Ritz BR. Increased epigenetic age and granulocyte counts in the blood of Parkinson's disease patients. Aging (Albany NY). 2015;7(12):1130-42.

22. Horvath S, Pirazzini C, Bacalini MG, Gentilini D, Di Blasio AM, Delledonne M, et al. Decreased epigenetic age of PBMCs from Italian semi-supercentenarians and their offspring. Aging (Albany NY). 2015;7(12):1159-70.

23. McEwen LM, Morin AM, Edgar RD, MacIsaac JL, Jones MJ, Dow WH, et al. Differential DNA methylation and lymphocyte proportions in a Costa Rican high longevity region. Epigenetics Chromatin. 2017;10:21.

24. Soriano-Tarraga C, Giralt-Steinhauer E, Mola-Caminal M, Vivanco-Hidalgo RM, Ois A, Rodriguez-Campello A, et al. Ischemic stroke patients are biologically older than their chronological age. Aging (Albany NY). 2016;8(11):2655-66.
